# Parasite spillover rather than niche expansion explains infection of host brain by diplostomid eye flukes

**DOI:** 10.1098/rspb.2024.2648

**Published:** 2025-02-05

**Authors:** Alfonso Diaz-Suarez, Veljo Kisand, Siim Kahar, Riho Gross, Anti Vasemägi, Kristina Noreikiene

**Affiliations:** ^1^ Chair of Aquaculture, Institute of Veterinary Medicine and Animal Sciences, Estonian University of Life Sciences, Kreutzwaldi 46, Tartu 51006, Estonia; ^2^ Institute of Technology, University of Tartu, Tartu 50090, Estonia; ^3^ Institute of Agricultural and Environmental Sciences, Estonian University of Life Sciences, Kreutzwaldi 5, Tartu 51006, Estonia; ^4^ Department of Aquatic Resources, Swedish University of Agricultural Sciences, Stångholmsvägen 2, Drottningholm 17893, Sweden; ^5^ Life Sciences Center, Institute of Biosciences, Vilnius University, Vilnius, Lithuania

**Keywords:** eye parasite, brain infection, *Perca fluviatilis*, DNA metabarcoding, tissue tropism, poolseq

## Abstract

Parasites often occupy specific sites within their host, which has important implications for host performance and parasite transmission. Nonetheless, parasitic infections can occur beyond their typical location within a host, significantly altering host–parasite interactions. Yet, the causes behind the atypical tissue tropism are poorly understood. Here, we focus on a ubiquitous group of diplostomid parasites that form diverse communities in fish eyes. We used targeted DNA metabarcoding (cytochrome c oxydase subunit 1, COX1, 250 bp) to evaluate potential mechanisms underlying eye parasite atypical tissue tropism to the brain of two widespread fish species (Eurasian perch and common roach). We found that the most common eye-infecting species (*Tylodelphys clavata*, *Diplostomum baeri*) are present in the brains of perch but not in roach. The bipartite network comprising 5 species and 24 mitochondrial haplotypes revealed no brain-specific haplotypes, indicating an apparent lack of genetic divergence between brain- and eye-infecting parasites. Instead, the prevalence, intensity and diversity of eye infections were positively correlated with brain infections. Thus, our results suggest that the most parsimonious mechanism underlying brain infection is density-dependent spillover rather than parasite divergence-driven niche expansion. We anticipate that ‘off-target’ infections are likely to be severely underestimated in nature with important ecological, evolutionary and medical implications.

## 1. Introduction

Most living organisms become infected with parasites at some point in their lives and serve as permanent or temporary habitat fragments within parasites’ life cycles. Often, an infected host carries an entire parasite metacommunity, and rules governing its assembly are thought to include a set of scale-dependent filters [1,2]. Thus, parasites show considerable variation in their ability to infect a specific host species or population, reflecting a long history of antagonistic host–parasite interactions [3]. In this context, parasites with complex life cycles are particularly intriguing because they must sequentially infect multiple hosts and each host represents a set of unique challenges as well as opportunities for evolutionary change [4]. Within a host, parasitic organisms also display a certain degree of microhabitat selectivity, also known as tissue tropism, because of preferential infection of specific sites within their host [5,6]. In many cases, this is the consequence of niche specialization resulting from both host–parasite and parasite–parasite interactions [7,8]. Effective tissue tropism plays an important role because the site of infection is strongly associated with the parasite’s ability to complete its life cycle [9]. Among all potential niches within a host, organs with limited immune response are especially attractive for a variety of parasite groups [10], and the specialization towards organs within the neurosensory system is known to improve the chances of transmission for many parasites with complex life cycles [11,12].

Despite the expected benefits of micro-niche specialization, parasites are often found beyond their typical infection sites [13]. For example, pathogenic protozoans, like *Trypanosoma brucei,* which causes African trypanosomiasis, or *Toxopasma gondii*, the causal agent of toxoplasmosis, are often found in atypical sites with important implications for disease symptoms and outcomes [14–16]. Similarly, the parasitic nematode *Onchocerca volvulus* typically found in the eyes or skin, causing ‘river blindness’, may also cross the brain–blood barrier, leading to neurological symptoms in the host [17]. Several mechanisms have been proposed to explain such ‘off-target’ infections. In some cases, parasite micro-niche preference appears to be mostly driven by the host species [18]. In other groups, parasite serotypes or lineages are important predictors of infections at atypical sites [19–21]. Furthermore, infection intensity often plays a key role in creating suitable conditions for within-host spillover to other tissues [22,23]. A more advanced understanding of key factors behind parasite micro-niche breadth and its potential expansion could provide important insights into host–parasite interaction and disease pathophysiology [24,25]. However, the mechanisms underlying micro-niche selectivity and atypical tropism are poorly understood for most parasite groups [1,25].

Diplostomids (Trematoda: *Digenea*) are a group of diverse and globally distributed parasitic flatworms that are ubiquitous in marine and freshwater ecosystems. They have a complex life cycle usually involving two intermediate hosts (snails and fish) and fish-eating birds as definitive hosts [26]. In fish, diplostomids are primarily found in the eyes, forming diverse communities consisting of multiple species and hundreds of individuals [27–29]. Typically, diplostomids can infect a wide variety of fish species [30] but also show some degree of host specialization [31]. Much stronger specialization is observed for microhabitat selection with different species occupying specific structures within the eye, such as the lens, vitreous humour and retina [32,33]. The consequences of intense infections in fish include impaired vision leading to reduced foraging efficiency and antipredator behaviours with negative effects on fish performance [34,35]. For example, severe infection with *Diplostomum spathaceum* reduces the growth of whitefish (*Coregonus lavaretus*) in experimental conditions. Similarly, infection with *Tylodelphys scheuringi* has been shown to decrease long-term growth of wild yellow perch (*Perca flavescens*) [36,37]. Furthermore, some diplostomid species can infect the brain. For example, two lineages of *Tylodelphys* sp. have been reported in the brain of silverside (*Chirostoma humboldtianum* and *C. jordani*) and the common bully (*Gobiomorphus cotidianus*) [38,39], whereas *Diplostomum* sp. lineage 4 has been shown to infect the brain of the three-spined stickleback (*Gasterosteus aculeatus*) and two lamprey species (*Lampetra fluviatilis* and *Lethenteron camtschaticum*) [40,41]. Yet, given that most diplostomid research has focused on host eye infections, this bias may have generated a gross underestimation of the occurrence and prevalence in other tissues.

A recently developed targeted metabarcoding approach allows fast screening of whole diplostomid communities in complex tissues such as the eyes [28]. Thus, this approach enables us to not only more comprehensively characterize parasite diversity but also reveal potential drivers of atypical tissue tropism. In this study, we used this approach to evaluate the prevalence and drivers of atypical tissue tropism of diplostomids in two widely distributed and abundant fish species, Eurasian perch (*Perca fluviatilis*) and common roach (*Rutilus rutilus*). We examined two potential mechanisms, namely (i) niche expansion and (ii) density-dependent colonization, as the underlying causes of diplostomid brain infection in fish. If brain infection is linked to niche expansion, we expect that the adaptation of the parasite to alternative tissues may have led to genetic divergence between eye- and brain-infecting genotypes [42]. Alternatively, if brain infection is a density-dependent process and can be viewed as within-host parasite spillover from typical to neighbouring tissue, we expect a lack of genetic divergence among parasites in different tissues, as well as a positive correlation between the prevalence, intensity, and haplotype diversity of eye (potential source) and brain (potential sink) infections. Our study reveals novel insights into the potential ecological and evolutionary consequences of tissue tropism in a ubiquitous and globally distributed group of parasites.

## 2. Material and methods

### (a) Fish sampling

Eurasian perch (*n* = 238, mean fork length (FL) = 144 mm, s.d. = 28.3) and common roach (*n* = 250, mean FL = 149 mm, s.d. = 15.4) were collected from seven lakes in Estonia in July 2020 as described in [28]. Fish were captured using gill nets submerged in the water for 1 h. Subsequently, fish were removed from the nets, kept cold during transport to the laboratory and stored at −20°C until further processing.

### (b) Fish processing and DNA isolation

Fish was weighed to the nearest 0.01 g, FL measured to the nearest mm and gonads were examined to determine sex. Since both species have a high prevalence of diplostomid eye infection in the sampled lakes [28], the brain was first dissected to prevent possible parasite contamination from the eyes to the brain during preparation. The fish was stabilized, the frontal portion of the skull was carefully removed with a scalpel, nerves were severed, the brain was cut at the distal end of the *medulla oblongata* and removed from the cranial cavity. Subsequently, both complete eyeballs were removed. To avoid cross-contamination, the brain and eyes were extracted using different dissection kits, and all dissection tools were alcohol-flame sterilized between individuals. Next, the brain and both eyeballs were digested in 4 ml of lysis buffer (0.4 M NaCl, 10 mM Tris-HCl, pH = 8, 2 mM EDTA, 2% sodium dodecyl sulfate) overnight at 56°C together with 0.4 ml of 20% SDS and 40 μl of Proteinase K (Thermo Scientific). The final lysis volume was increased to 8 ml for larger fish (FL > 180 mm). DNA isolation was performed from the resulting lysate following a standard salt extraction method [43]. The concentration of isolated DNA was quantified using Nanodrop 2000 (Thermo Scientific).

### (c) Brain infection prevalence

To determine whether diplostomid DNA was present in perch and roach brain tissue, a randomly selected subset of DNA samples isolated from the brain lysate (*n* = 84, 12 individuals of each species per lake) was screened using endpoint polymerase chain reaction (PCR) amplification and agarose gel electrophoresis. A cytochrome *c* oxidase subunit 1 fragment (COX1) was amplified using Plat-diploCOX1 diplostomid-specific primer [44]. Amplification was performed in a final volume of 10 µl using 2 µl Hot Firepol^®^ Blend Master mix (Solis Biodyne), 2 µl of total DNA, 5 pmol of each primer and 10 pmol of MgCl_2_. PCR conditions consisted of a 15 min initial activation at 95°C, followed by 30 cycles of denaturation for 20 s at 95°C, annealing for 30 s at 58°C, a 30 s extension at 72°C and a final 10 min extension at 72°C. PCR products were visualized on 1% agarose gel and indicated that brain infection was present only in perch (27 perch out of 84 showed positive amplification).

To obtain an independent confirmation that live diplostomid parasites are present in the brain of perch, 30 additional perch specimens were collected from one of the studied lakes (Saadjärv) in collaboration with local fishermen. Freshly caught fish were processed as previously described, but the brain and cranial cavity were examined under a stereomicroscope (Leica EZ4D). In three specimens, live diplostomid metacercariae were observed moving freely throughout the brain beneath the neurocranium. The detected metacercariae were individually collected and stored in 96% ethanol until DNA extraction using the DNAeasy^®^ blood and tissue kit (Qiagen). The same COX1 fragment (*ca* 500 bp) was amplified from the DNA isolated from single fluke parasites as described previously, and Sanger sequencing was performed in both directions at the core facility of the Institute of Genomics (Tartu, Estonia). Forward and reverse sequences were concatenated using SeqTrace [45], and species identity for four fluke specimens was established using Basic Local Allignment Search Tool (BLAST) with 100% coverage and ≥ 97% similarity threshold [46].

#### (i) Library preparation

To characterize the diplostomid communities in the brain and eyes of perch, we prepared quadruple-indexed libraries using a dual PCR method, which allows for cost-efficient labelling of a large number of samples [47]. The library included 251 DNA samples isolated from the eyes and brain lysates of 181 perch. During the first PCR, a COX1 fragment was amplified with Plat-diploCOX1 diplostomid-specific primers modified with internal tagging indexes [47] (electronic supplementary material, table S1). PCR was performed using 2× QMP reagent (QIAGEN Multiplex PCR Kit) in a final volume of 10 µl, 3 pmol of each primer and approximately 100 ng of total DNA. PCR conditions consisted of an initial activation for 15 min at 95°C, followed by 35 cycles of denaturation for 30 s at 94°C, annealing for 90 s at 57°C, a 90 s extension at 72°C and a final 10 min extension at 72°C. During the second PCR, the external indexes and Illumina sequencing adapters were incorporated into the PCR product of the first reaction using limited-cycle PCR (electronic supplementary material, table S2). The second PCR included 2.4 µl of purified water, 3 pmol of each primer, 5 µl of 2× QMP reagent (QIAGEN Multiplex PCR Kit) and 2 µl of the previous PCR product in a final volume of 10 µl. The limited-cycle PCR conditions consisted of an initial activation for 15 min at 95°C, followed by 15 cycles of denaturation for 30 s at 94°C, annealing for 90 s at 60°C, a 90 s extension at 72°C and a final 10 min extension at 72°C. To reduce false positives and increase diversity detection during PCR amplification [48], each DNA sample was amplified twice, and the product from each first PCR was subsequently used as the starting material for the second amplification, incorporating different external indexes into each PCR product. Consequently, each PCR replicate was tagged with a unique index combination. In addition, four negative controls using RNase-free water (Qiagen) were included in each 96 well plate to quantify the extent of Illumina index hopping [49]. All libraries were pooled and purified using AMPure XP beads (Beckman). After cleaning and size selection, libraries were sequenced (paired-end sequencing with 2 × 300 bp read length) using an Illumina MiSeq instrument (Illumina Inc., San Diego, CA, USA) at the Uppsala Biomedical Centre (SciLifeLab, Uppsala, Sweden).

#### (ii) Bioinformatic pipeline

The 300 bp paired-end reads generated with the quadrupled indexed library were processed at UPPMAX (Uppsala Multidisciplinary Center for Advanced Computational Sciences, Sweden). The internal indexes and diplostomid-specific primers incorporated during the first PCR were demultiplexed and trimmed using cutadapt v. 3.1 [50]. Pair merging was attempted with PEAR v. 0.9.10 [51], but the low quality of the reverse sequences resulted in a small number of retained reads (0.02%). Therefore, only forward reads were considered for subsequent analysis. Also, to increase the number of retained sequences, 50 bp were trimmed from the 3′ end using cutadapt v. 3.1. The resulting 250 bp reads were quality filtered with VSEARCH v. 2.18.0 [52] using the fastx_filter function with a maximum expected error of 1 (fastq_maxee 1). The amplicon sequence variants (ASVs) determination, denoising, dereplication and chimera filtering were performed using the functions derep_fulllength, cluster_unoise and uchime3 functions, respectively, using VSEARCH. ASVs were used because they provide higher resolution and accuracy in describing genetic diversity compared with clustering methods [53]. The taxonomic classification of ASVs was performed with SINTAX classifier [54] with a minimum bootstrap support of 90% and a custom database created with CRABS [55], including all Diplostomidae COX1 sequences available in National Center for Biotechnology Information (NCBI) as of July 2023.

#### (iii) Statistical analysis

As a first step, reads were normalized using their mean sequencing depth. This was performed by dividing the number of reads of every ASV by the total number of reads of that sample and multiplying by the mean number of reads of the entire dataset. To prevent false positives, only the ASVs allocated to both PCR replicates were considered true positives [48]. Additionally, a maximum contamination threshold was established, turning into zero any read counts below or equal to the maximum read count (*n* = 4) of the most common ASV within a negative control [56]. From this point, the remaining ASVs are considered as haplotypes and they were mapped to the samples. The resulting data matrix was analysed in the R computing environment [57].

A binary presence/absence matrix was constructed for all identified haplotypes to assemble a bipartite network using the bipartite v. 2.18 package [58]. A bipartite network consists of two different types of nodes, and edges can only connect nodes of different types. In this case, one of the nodes included 24 haplotypes across 5 detected parasite species, and the second node corresponded to the site of infection (brain and eye). Links between the two types of nodes were established if a haplotype was observed in a tissue, and the weight of the lines represents the total number of observations of that haplotype in the tissue among all studied fish. Next, to determine the odds of brain infection relative to eye infection, odds ratios and 95% confidence intervals (CIs) were calculated with the Haldane-Anscombe correction for each *Tylodelphys clavata* and *Diplostomum baeri* haplotype [59,60]. Additionally, for the two most common and genetically diverse diplostomid parasite species (*T. clavata* and *D. baeri*), we constructed parsimonious haplotype networks using the pegas v. 1.2 package [61] to better understand the distribution and frequency of the observed haplotypes among studied lakes.

To characterize the relationship between eye and brain infection, we calculated Spearman correlations between the eye and brain prevalence of *T. clavata* and *D. baeri* in all studied lakes. Furthermore, we used linear regression to test the association between the square root transformation of the number of sequence reads assigned to the eyes and brain for both parasite species using the stats package v. 4.1.3 [57].

To identify factors that influence the probability of brain infection, we used a general linear model (GLM) with a bivariate response variable (1 = infected, 0 = uninfected) and binomial error distribution. Predictors included in the model were as follows: (i) lake (fixed factor with seven levels and randomly chosen reference level (lake = Hino)) to account for location-dependent differences, (ii) natural logarithm transformation of the FL (ln (FL)) as a proxy for age to control for the potential accumulation of parasites throughout the life of the fish [29], (iii) square root transformation of the number of parasite reads in the eye as a proxy of the infection intensity and (iv) the number of distinct parasite haplotypes in the eye. The significant effect of the last two factors would indicate that diplostomid infection in the two tissues is interconnected, and most likely can be explained by the parasite spillover process, where infection rates and haplotype diversity in the eye and brain show a positive correlation.

To further reveal the factors influencing diplostomid diversity in the brain, we also constructed two GLMs with Poisson and negative binomial distributions, respectively, where the response variable was the number of parasite haplotypes detected in the brain using the same set of predictors as described above.

To investigate the potential relationship between diplostomid infection rate and individual variation in host body condition, we built a linear model (LM) with condition factor (K_c_) as the response variable. K_c_ was calculated as 100 × W/FL^3^, where W represents weight in grams and FL represents FL in centimetres. In this LM, the predictors were as follows: (i) lake (fixed factor with seven levels), (ii) the number of *T. clavata* haplotypes in the eye (hapTyl eye), (iii) the number of *D. baeri* haplotypes in the eye (hapBae eye), (iv) the number of haplotypes of *D. spathaceum*, *D. pseudospathaceum* and *D. rauschi* in the eye (hapOth eye), (v) the number of *T. clavata* haplotypes in the brain (hapTyl brain) and (vi) the number of *D. baeri* haplotypes in the brain (hapBae brain).

All three models were run using functions within the stats v. 4.1.3 package [57]. For the three response variables, several models were assembled using different combinations of the predictors, and the best resulting model for each response variable was determined according to Akaike‘s information criterion (AICc) using the AICcmodavg v. 2.3.2 package [62]. The collinearity among predictors of the constructed models was determined using the variance inflation factor calculated using the performance v. 0.10.5 package [63]. Model diagnostic plots were visually inspected for influential data points, and homoscedasticity of residuals and model results were plotted using functions within ggplot2 v. 3.4.2 [64].

## 3. Results

### (a) Host-specific prevalence of diplostomid infection in the brain

Initial endpoint PCR screening of sympatric perch (*n* = 84) and roach (*n* = 84) collected from seven lakes indicated that diplostomid parasite DNA was present only in the brain of perch. Visual inspection of the 30 additional freshly caught perch confirmed the presence of live and freely moving diplostomid parasites in at least three fish, with the number of live flukes detected ranging from one to six per infected specimen. All sequenced individual flukes (*n* = 4) were identified as *T. clavata* (sequence similarity 99.8% for 474−478 bp).

### (b) DNA metabarcoding and diplostomid diversity

Over 19 million raw reads were generated across all brain and eye samples. Of these reads, 992 983 were retained after demultiplexing, primer trimming and quality filtering, resulting in 24 ASVs (haplotypes) from five diplostomid species. Altogether, 205 995 reads were mapped to the samples (mean number of sequences mapped per replicate = 838.3, electronic supplementary material, figure S1). Most of the diplostomid reads were classified as *T. clavata* (96.11%) and *D. baeri* (3.52%), whereas *D. pseudospathaceum* (0.17%), *D. rauschi* (0.14%) and *D. spathaceum* (0.03%) were less abundant (electronic supplementary material figure S2). Among the identified 24 ASVs (haplotypes), 7 belonged to *T. clavata* and 11 to *D. baeri*, while the number of detected haplotypes for other species was lower (*D. rauschi* = 3, *D. pseudospathaceum* = 2*, D. spathaceum* = 1) (electronic supplementary material, table S3). Based on 18 negative control samples, limited index hopping was detected, as we observed a maximum of four assigned reads in negative control samples. As expected, all ASVs detected in the negative control samples were identified as the most common ASV, corresponding to *T. clavata* haplotype 1. Therefore, we considered all samples with four or fewer reads of *T. clavata* haplotype 1 as non-infected. In addition, the PCR replicates showed similar results (Pearson correlation *R*^2^ = 0.712, *p* < 0.0001), suggesting a low rate of technical variation in our dataset.

### (c) Species prevalence and tissue tropism

In total, five diplostomid species were detected among perch eye samples. *T. clavata* showed the highest prevalence (87.2%) followed by *D. baeri* (46.4%), while for the three remaining species, the prevalence estimates were <2% (table 1). The overall eye infection prevalence varied from 50 to 100% (average 90.1%), reaching 100% in three lakes (electronic supplementary material, table S4). The two most common eye parasites, *T. clavata* and *D. baeri,* were also detected in perch brain. The prevalence of brain infections ranged from 0 to 46.2% (average 31.5%). All brain infections were caused by *T. clavata* (31.7%), while the prevalence of *D. baeri* was considerably lower (2%) and always co-occurred with *T. clavata*. All perch specimens (*n* = 56) showing signals of diplostomid infection in the brain also showed signals in the eye, except for a single specimen (table 1).

**Table 1.** Number of infected perch (prevalence, %) with the five parasite species in each lake and tissue.

	prevalence										
		*T. clavata*		*D. baeri*		*D. pseudospathaceum*		*D. spathaceum*		*D. rauschi*	
lake	*n*	eye	brain	eye	brain	eye	brain	eye	brain	eye	brain
Hino	20	8 (40%)	0	3 (15%)	0	0	0	0	0	0	0
Kasaritsa Verijärv	31	30 (97%)	13 (41%)	0	0	0	0	0	0	0	0
Kisõjärv	19	10 (55.6%)	1 (5.6%)	8 (44.4%)	0	0	0	1 (5.6%)	0	0	0
Koorküla Valgjärv	19	14 (87.5%)	2 (12.5%)	1 (6.2%)	0	0	0	0	0	0	0
Õisu	31	30 (100%)	11 (36.7%)	25 (83.3%)	2 (6.7%)	3 (10%)	0		0	1 (3.3%)	0
Piigandi	27	27 (100%)	12 (44.4%)	21 (77.7%)	1 (3.7%)	0	0	1 (3.7%)	0	0	0
Saadjärv	39	39 (100%)	18 (46.2%)	26 (66.6%)	1 (2.6%)	0	0		0	1 (2.6%)	0
total/average	181	158 (87.3%)	57 (31.5%)	84 (46.4%)	4 (2.2%)	3 (1.7%)	0	2 (1.1%)	0	2 (1.1%)	0

**Figure 1.**
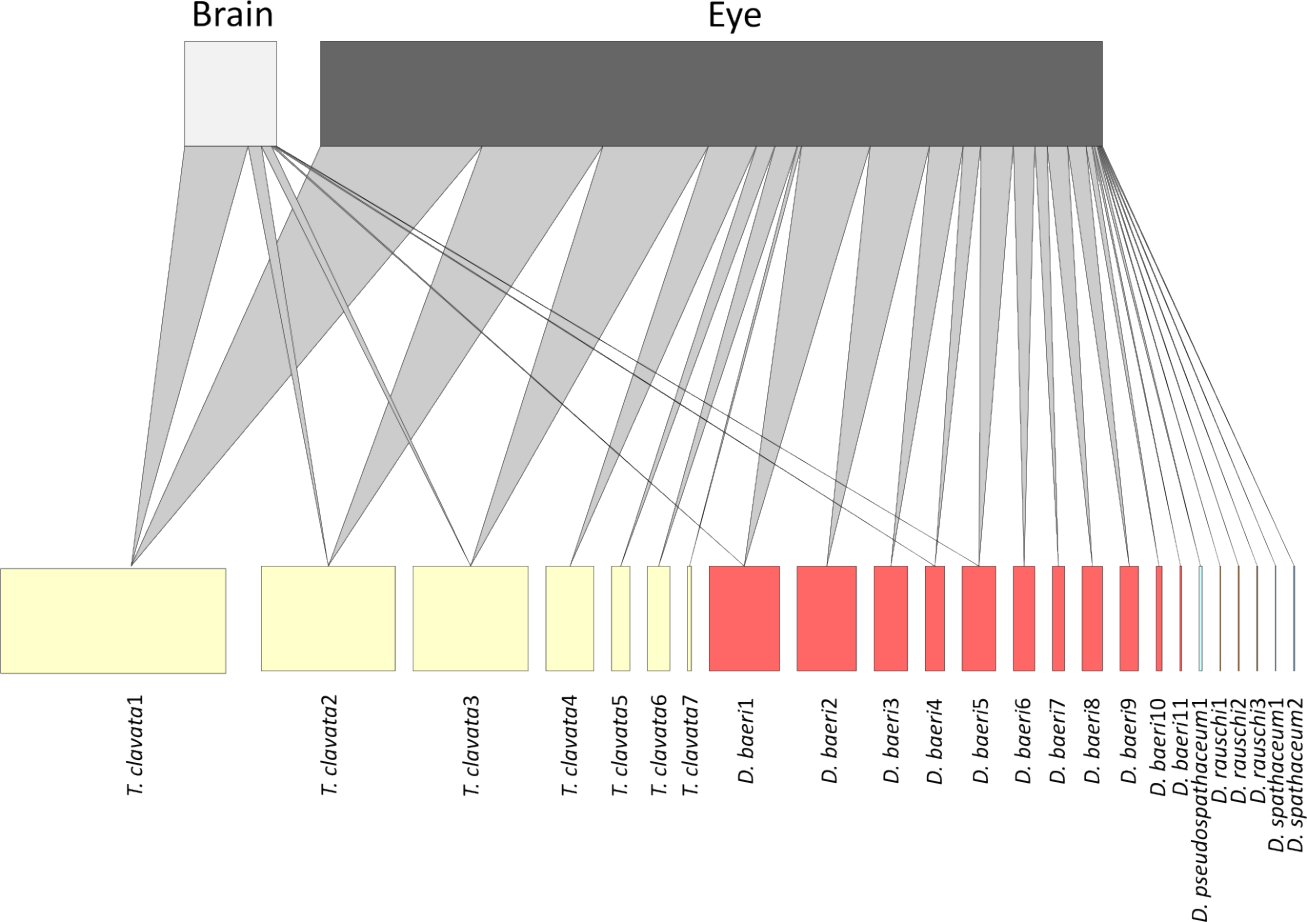
Bipartite network plot of diplostomid parasite species found in the brain and eyes of perch with 24 ASVs (haplotypes) belonging to five parasite species as one of the edges (bottom) and two potential infection sites within the host as the other edge (top). Links between the two types of nodes were established if a haplotype was observed in a tissue, and the weight of the lines is proportional to the total number of observations of that haplotype in the tissue among all studied fish.

The constructed bipartite network based on 24 haplotypes of the five parasite species revealed no brain-specific haplotypes (figure 1). Three of the seven *T. clavata* haplotypes were found in the brain, corresponding to the most common variants. Similarly, 3 of the 11 haplotypes of *D. baeri* were detected in the brain (figure 1). The odds ratios calculated for *T. clavata* and *D. baeri* haplotypes ranged from 0.0006 to 0.106 (average 0.046), but their CIs largely overlapped (electronic supplementary material, table S5).

### (d) Lack of intraspecific structuring among studied lakes

**Figure 2.**
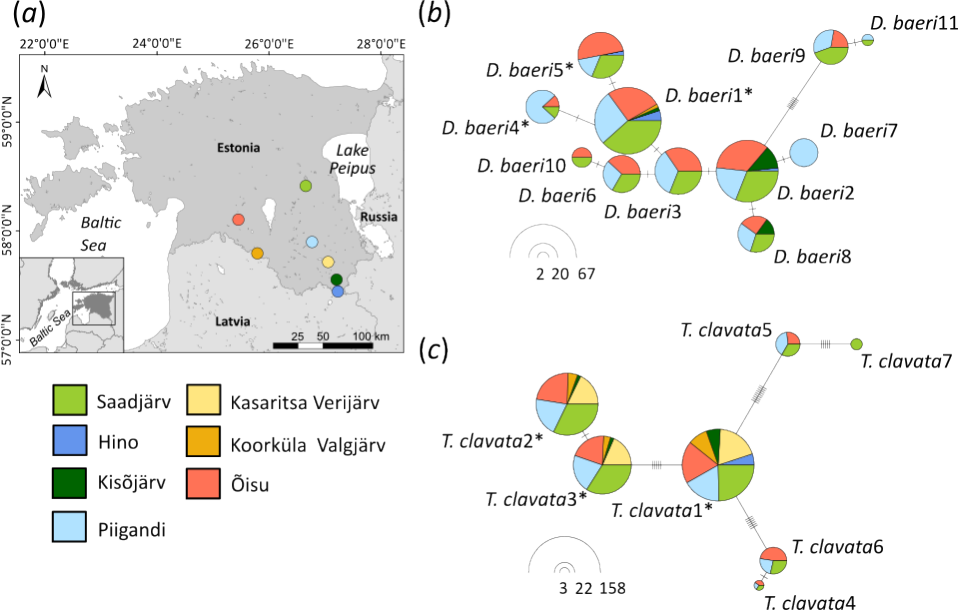
Sampled locations (*a*) and haplotype networks for the two most common diplostomid parasite species in perch: (*b*) *D. baeri* and (*c*) *T. clavata* across seven lakes in Estonia. The circle area represents the number of fish infected by each haplotype, and haplotypes found in eye and brain are marked with an asterisk (*). The small black lines represent the number of mutations between haplotypes. The different colours represent the proportion of each haplotype recovered in the seven lakes.

The haplotype network analysis revealed no evidence of spatial structuring for the two most frequent parasite species (figure 2). *T. clavata* was dominated by a common haplotype (*T. clavata*1) occurring at a frequency of 87.3% in all studied lakes. Furthermore, the two other haplotypes occurred in six of the seven lakes, and only one rare haplotype was found in Lake Saadjärv. For *D. baeri*, two common haplotypes were detected with frequencies of 37.0% (*D. baeri*1) and 32.0% (*D. baeri*2) present in all but one (Kasaritsa Verijärv) and two lakes (Kasaritsa Verijärv and Koorküla Valgjärv), respectively. The maximum difference between the two haplotypes in *T. clavata* and *D. baeri* was 22 and 12 mutations, respectively.

### (e) Relationships between eye and brain infections

**Figure 3.**
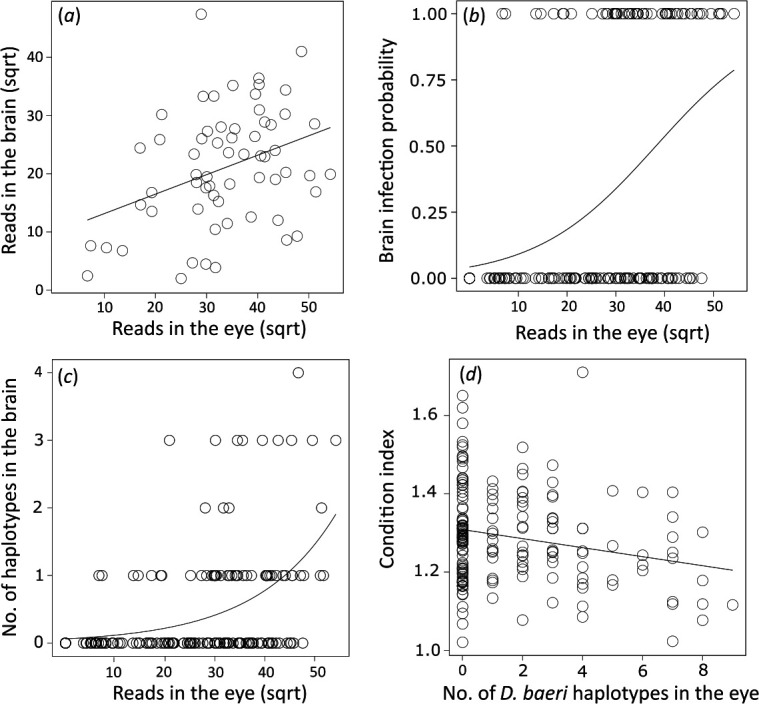
Analyses of relationships between eye and brain infections: (*a*) simple linear regression between the number of reads assigned to *T. clavata* in the brain and eyes of the studied perch, (*b*) estimation of the diplostomid brain infection probability (1 = infected, 0 = uninfected) based on the number of reads assigned to the eye. Prediction was calculated from the best general linear model (GLM) according to the corrected Akaike‘s information criterion, (*c*) estimation of the number of haplotypes in the brain associated with the number of reads assigned to the eye. Prediction was calculated from the best GLM selected according to the corrected Akaike‘s information criterion, (*d*) estimation of perch body condition based on the *D. baeri* diversity in the eye. Prediction was calculated from the best linear model (LM) according to the corrected Akaike‘s information criterion. Square root transformation (sqrt).

Spearman correlation analyses indicated a positive association between eye and brain prevalence in the seven lakes for both *T. clavata* (*r_s_* = 0.89, *p* = 0.007) and *D. baeri* (*r_s_* = 0.94, *p* ≤ 0.001). At the individual level, linear regression revealed a positive correlation in the number of *T. clavata* sequences between the eye and brain (*R*^2^ = 0.13, *p* = 0.004) (figure 3*a*). The AICc indicated that the best model explaining the probability of brain infection as a response variable includes only the number of reads assigned to the eye as a predictor (electronic supplementary material, table S6). The number of parasite sequence reads in the eye was also positively associated with the probability of brain infection (*R*^2^ = 0.192, s.e. = 0.015, 95% CI (1.05−1.12), *p* ≤ 0.0001, figure 3*b*), and none of the remaining predictors had a significant effect (table 2). A similar result was obtained using GLM considering the number of haplotypes in the brain as the response variable (electronic supplementary material, table S9), which showed a significant positive association (*R*^2^ = 0.352, s.e. = 0.009, 95% CI (1.05−1.09), *p* ≤ 0.0001, figure 3*c*) with the number of reads in the eye (table 2). The best LM according to AICc considering condition factor K_c_ as the response variable included lake and the number of *D. baeri* haplotypes, but not *T. clavata* haplotypes in the eye (electronic supplementary material, table S12). Also, a negative association between fish condition and haplotype diversity of *D. baeri* was observed (*R*^2^ = 0.244, s.e. = 0.004, 95% CI (−0.02 to 0.00), *p* = 0.004; figure 3*d*, table 3).

**Table 2.** The probability of diplostomid brain infection and the diversity of the brain infection in perch based on the two best general linear models (GLM) according to the corrected Akaike‘s information criterion (AIC). Reads eye indicates the number of reads assigned to the eye.

predictors	estimate	standard error	Z value	*p*-value
response variable: brain infection				
intercept	−3.1185	0.5228	−5.964	<0.001
reads eye	0.0815	0.015	5.212	<0.001
response variable: number of haplotypes in the brain				
intercept	−2.7715	0.3784	−7.323	<0.001
reads eye	0.0630	0.0099	6.315	<0.001

**Table 3.** Results of the best linear model (LM) according to the corrected Akaike‘s information criterion (AIC) of the predictors determining perch condition factor K_c_ infected by diplostomid parasites. HapBae eye indicates the number of *Diplostomum baeri* haplotypes in the eye.

response variable: condition factor (K_c_)				
predictors	estimate	standard error	Z value	*p*-value
intercept	1.2053	0.0239	50.353	<0.001
lake: Kasaritsa Verijärv	1.6682	0.0311	5.364	<0.001
lake: Kisõjärv	0.1505	0.0348	4.321	<0.001
lake: Koorküla Valgjärv	0.0587	0.0373	1.576	0.116
lake: Õisu	0.1556	0.0331	4.693	<0.001
lake: Piigandi	0.08811	0.0342	2.575	0.0108
lake: Saadjärv	0.08154	0.3071	2.655	0.0086
HapBae eye	−0.0127	0.0043	−2.926	0.003

## 4. Discussion

The success of a parasite with a complex life cycle rests on its ability to overcome multiple scale-dependent filters and reach the next susceptible host within an optimal time frame [65,66]. Effective within-host tissue tropism plays a pivotal role in maximizing the chances of survival and transmission through immune evasion, resource use and even host manipulation [11,12]. Why then infections occur seemingly at non-target sites, although outcomes may be unpredictable for both the host and parasite, is an open question in medical and evolutionary fields [67]. Here, we tested two alternative hypotheses to explain previously undescribed atypical brain tropism by diplostomid parasites in common freshwater fish species. By harnessing the power of a targeted metabarcoding approach, we found evidence for density-dependent colonization, rather than haplotype-specific niche expansion, explaining diplostomid eye fluke infections in the brain of Eurasian perch. In contrast, brains of common roach were found to be free of diplostomids, despite harbouring similar parasite communities in the eye. The observed high prevalence of brain infections in perch demonstrates that even for relatively well-studied parasite groups and widely distributed host species, atypical tropisms are likely severely under-reported, highlighting the potentially overlooked consequences of cryptic ‘off-target’ infections in the wild.

Atypical tissue tropism is a common phenomenon in many parasite groups, but the underlying mechanisms may differ depending on the investigated host–parasite system [18–20]. In this study, we tested two hypotheses, niche expansion and density-dependent colonization, as alternative explanations for fish brain infection by diplostomids [6,28,30]. Based on multiple lines of evidence, we found strong support for density-dependent colonization of perch brains by diplostomids. First, at the host population level, brain infections occurred in all investigated lakes except one, and the prevalence of brain and eye infections was positively correlated. Second, at the host level, nearly all brain infections co-occurred with eye infections. Third, the probability of brain infection, as well as the number of parasite haplotypes detected in the brain, were positively associated with the intensity of eye infection reflected by parasite read number. Finally, bipartite network analysis showed that none of the 24 haplotypes from 5 diplostomid species were brain specific, and the calculated odds ratios indicated that all haplotypes have rather similar probabilities of infecting the brain. Thus, multiple lines of evidence suggest that density dependence is the most parsimonious explanation for the occurrence of atypical brain tropism of diplostomid parasites in perch [68–70]. Similar density-dependent patterns have been documented in several host–parasite systems, indicating that this might be a common mechanism underlying ‘off-target’ infection [22,23,71].

In this study, we detected five diplostomid species in the investigated perch tissues. The most common were *T. clavata* and *D. baeri,* which were also found infecting perch brains. Both species are typically located in the vitreous humour of the eye where flukes move freely. Thus, unlike diplostomids specializing in infecting lenses [72,73], freely moving diplostomids may have higher chances of reaching new tissues such as the brain. Especially because both species also reach very high infection intensities [34,35]. However, due to the apparent ability to migrate out of the eye during the entire duration of infection, the observed density-dependent patterns may result from two mutually non-exclusive temporal scenarios, due to chronic within-host spillover or acute exposure to diplostomid cercariae. Based on the first scenario, density dependence is a consequence of a source-sink system [74] and parasites migrate initially to the eye and then, to the brain. Alternatively, when many cercariae are released from the snail (first intermediate host) to infect fish, some parasites may haphazardly reach the brain in a density-dependent manner and survive due to the limited immune response [10]. However, very little is known about the exact mechanisms of tissue tropism in most trematodes and diplostomids [75,76] and without further experimental work, it is not possible to distinguish which infection scenarios are primarily responsible for the observed density-dependent patterns.

Although diplostomids are known to impair the vision of their host, hampering prey detection, feeding efficiency and survival of infected fish [35,36], previous studies on the effect of parasite infection on fish body conditions have shown mixed effects. For example, a strong negative correlation between body condition and parasite load was found in only one of three Antarctic fish species [77], while the parasite effects on the body condition of common bullies range from positive to negative depending on parasite species and infection intensity [78]. Consistent with this, our results reveal a weak but significant negative relationship between body condition and *D. baeri* but not *T. clavata* diversity in the perch eyes. These mixed results could be partly a consequence of the distinct effect of different eye fluke species, their abundance and developmental stages, as well as temporal and environmental effects [79]. Future studies should apply other methods such as blood biomarkers to determine the effect of diplostomid parasite infection and the location of infection on fish body condition [80].

Tissue tropism described here differs not only among parasite species but also between hosts. Typically, diplostomids are considered generalists that can infect a wide range of fish species [30]. According to earlier analyses of the same lakes, the eyes of perch and roach were largely infected by the same parasite species and haplotypes and showed only small differences in the diplostomid communities [28]. However, in this study, diplostomid brain infections were present only in perch. This could be, in part, a consequence of differences in host–parasite interaction between fish species. Although both hosts occupy similar habitats, perch and roach are phylogenetically distant and belong to different families [81]. Differences in sensitivity to parasite infection and the resulting pathologies are commonly observed between distant species [77,78]. Sometimes differences in parasite susceptibility are also observed for host species from the same family or even genus. For instance, salmonids are often infected by the myxozoan parasite *Tetracapsuloides bryosalmonae*, but sympatric Atlantic salmon (*Salmo salar*) and brown trout (*Salmo truta*) differ in their response [82]. Therefore, the immune defence system of roach may limit atypical tissue tropism to the brain. Differences in immune system repertoire between percids and cyprinids are expected due to the relatively large evolutionary distance [81]. However currently, detailed characterization of possible key components is missing.

Comparable to the growing number of studies, our work illustrates the power of metabarcoding for characterizing cryptic or poorly known parasite communities [83,84]. However, it also has some limitations. For example, extrapolating sequence read counts to infer biomass or individual specimen numbers is not straightforward and should be performed with caution [85,86]. Size differences between taxa, such as those observed among the diplostomid species detected in this study (*T. clavata* is larger than the remaining species)*,* could lead to variation in the relative concentration of DNA in pooled samples and affect the number of generated reads [29,87]. While here, we cannot provide direct evidence on the association between read number and individual parasite number, similar metabarcoding studies provide accumulating evidence of the strong correlation between read number and biomass/individual number in several taxa [88,89]. For more accurate molecular quantification of infecting parasites, further studies should test complementary methodologies, such as quantitative PCR, which have proved to be effective in quantifying parasite load in a wide range of species, such as *T. gondii, Borrelia burgdorferi* and *T. bryosalmonae* [90–92]. Furthermore, advancements in genomic approaches are increasingly expanding our ability to investigate interindividual differences [93]. This could also contribute to elucidating more subtle divergence at the intraspecific level for diplostomids. While sensitive and reliable [28,83,94,95], the metabarcoding approach based on short DNA sequence variation has limited capacity to characterize intraspecific diversity. In this study, we used only a small fragment (250 bp) from the mitochondrial genome [96,97] and found no genetic divergence between brain- and eye-infecting parasites. However, we cannot exclude genetic differences that exist in other regions of mitochondrial or nuclear genomes. Previous studies have shown genetic variation associated with tissue tropism in important pathogens. For example, whole-genome analysis revealed a genetic divergence between skin- and throat-infecting Streptococcus Group A, whereas two genetically distinct strains of *T. brucei* showed differences in tissue tropism and the associated pathology [19,98]. Similarly, *T. cruzi*, the parasitic protozoan that causes Chagas disease, is subdivided into six discrete typing units, which correspond to distinct COX1 lineages showing differences in tissue tropism, histopathology and disease symptoms [99,100]. Therefore, with the current metabarcoding data, we cannot entirely exclude the occurrence of genetic differentiation between brain- and eye-infecting parasites, and further studies will require the analysis of larger portions of the mitochondrial and nuclear genome. The application of genomic approaches for non-model species will promote the study of essential interactions in lesser-known host–parasite systems where the greatest diversity of evolutionary outcomes is expected [101].

Similar to diplostomid infections in the eye, parasites in the host brain may have important effects on the performance and behaviour of perch. Host manipulation is a common strategy in trophically transmitted parasites and is well-documented in diplostomids. For instance, eye infection by *D. spathaceum* reduces feeding efficiency and antipredator behaviour competence in rainbow trout [102]. Similarly, brain infection by the trematode *Euhaplorchis californiensis* in Californian killifish increases surface swing, which ultimately enhances the likelihood of predation by the final avian host [103]. Significant effects on perch performance have also been described for *T. clavata*, the species that most commonly infects the brain of the studied perch [34,35]. However, it is unclear whether these effects are caused by eye infection alone or by a combination of brain and eye infection, as the manipulation mechanism of ocular flukes does not depend exclusively on the deterioration of fish vision [104]. Infection of the central neural system is common in manipulative parasite species [105]. Therefore, the observed diplostomid brain infection has a high potential to affect the host. However, host manipulation is a complex process and should be carefully evaluated in the future [12].

## 5. Conclusions

We have demonstrated for the first time that ‘off-target’ diplostomid brain infections in Eurasian perch are a common phenomenon consistent with a density-dependent colonization process. Yet, more work is needed to elucidate the specific infection routes responsible for the observed infection patterns and consequences of brain infections at physiological or behavioural levels. Our work also indicates that ‘off-target’ infections are likely to be severely underestimated in nature with potential ecological and evolutionary consequences, as well as medical implications, for both host and parasite. We expect that future metabarcoding efforts will substantially increase our knowledge of atypical tissue tropisms in a wide range of host–parasite systems. Consequently, this will help to provide a better resolution for the underscored role of parasites in ecosystems.

## Data Availability

DNA sequences: GenBank accession nos. PP231874-PP231877. Data and code are available in Dryad [106]. The raw Illumina amplicon reads are available in the NCBI BioProject database with access number PRJNA1078141. Supplementary material is available online [107].
